# Yil102c-A is a Functional Homologue of the DPMII Subunit of Dolichyl Phosphate Mannose Synthase in *Saccharomyces cerevisiae*

**DOI:** 10.3390/ijms21238938

**Published:** 2020-11-25

**Authors:** Sebastian Piłsyk, Urszula Perlinska-Lenart, Anna Janik, Elżbieta Gryz, Marta Ajchler-Adamska, Joanna S. Kruszewska

**Affiliations:** Institute of Biochemistry and Biophysics Polish Academy of Sciences, 02-106 Warsaw, Poland; seba@ibb.waw.pl (S.P.); lenart@ibb.waw.pl (U.P.-L.); annaj@ibb.waw.pl (A.J.); egryz@ibb.waw.pl (E.G.); Marta.Ajchler@gmail.com (M.A.-A.)

**Keywords:** *Trichoderma reesei*, DPM2, GPI-GnT

## Abstract

In a wide range of organisms, dolichyl phosphate mannose (DPM) synthase is a complex of tree proteins Dpm1, Dpm2, and Dpm3. However, in the yeast *Saccharomyces cerevisiae*, it is believed to be a single Dpm1 protein. The function of Dpm3 is performed in *S. cerevisiae* by the C-terminal transmembrane domain of the catalytic subunit Dpm1. Until present, the regulatory Dpm2 protein has not been found in *S. cerevisiae*. In this study, we show that, in fact, the Yil102c-A protein interacts directly with Dpm1 in *S. cerevisiae* and influences its DPM synthase activity. Deletion of the *YIL102c-A* gene is lethal, and this phenotype is reversed by the *dpm2* gene from *Trichoderma reesei*. Functional analysis of Yil102c-A revealed that it also interacts with glucosylphosphatidylinositol-*N*-acetylglucosaminyl transferase (GPI-GnT), similar to DPM2 in human cells. Taken together, these results show that Yil102c-A is a functional homolog of DPMII from *T. reesei* and DPM2 from humans.

## 1. Introduction 

Dolichyl phosphate mannose (DPM) is the donor of four mannosyl residues in the oligosaccharide precursor formed on dolichyl phosphate (DolP) during protein *N*-glycosylation of the first mannose in *O*-mannosylation, of all three mannoses in glycosyl phosphatidyl inositols (GPIs) [1,2,3,4,5,6], and of the mannose in *C*-mannosylation [7]. 

**Figure 1:**
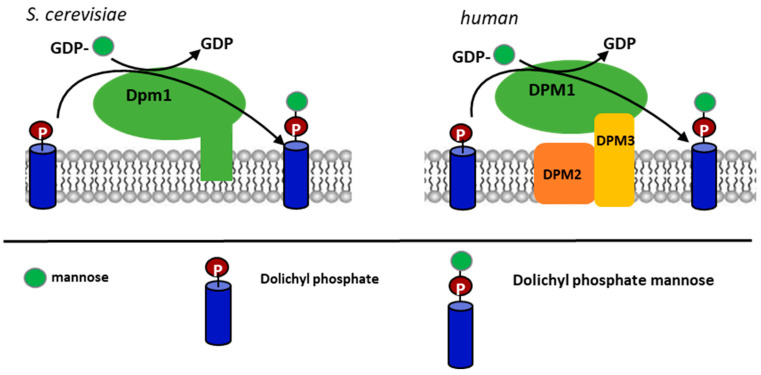
Schematic presentation of the dolichyl phosphate mannose (DPM) synthase structure from *S. cerevisiae* and the human group of the enzymes.

The synthesis of DPM is catalyzed by the DPM synthase, which transfers the mannosyl residue from (Guanosine Diphosphate –mannose) GDP-mannose to DolP. The *DPM1* gene encoding the DPM synthase in *S. cerevisiae* and the protein have been characterized [8,9], and homologous genes from *Ustilago maydis* and *Trypanosoma brucei* have also been reported [10,11]. The homologs from humans, *Schizosacchromyces pombe* and *Trichoderma reesei* [12,13], were found to form a separate class of the enzyme lacking the C-terminal transmembrane domain present in *S. cerevisiae*, *U. maydis*, and *T. brucei* synthases (Figure 1). Maeda et al. [14] reported that the DPM synthase from humans forms a complex of three subunits, DPM1, DPM2 and DPM3, where DPM1 is the catalytic subunit, DPM2 regulates its activity, and DPM3 anchors the complex in the ER membrane. A lack of the DPM2 subunit decreased the activity of the DPM synthase tenfold. On the other hand, it turned out that the Dpm2 subunit is not essential to compensate for a deletion of the *DPM1* gene in *S. cerevisiae* as a heterologous expression of the DPMI and DPMIII subunits from *T. reesei* in an *S. cerevisiae dpm1∆* mutant was sufficient to suppress the mutation [15]. In general, it has been established that if a Dpm1 protein lacks a transmembrane domain, it cannot attach to the ER membrane and—to be functional—needs the anchoring Dpm3 protein [12,13,16,17]. Ashida et al. [17] found that in the absence of DPM3, the DPM1 protein was rapidly degraded by the proteasome. In short, DPM3 determines the correct location of the catalytic subunit, and DPM2 is important but not critical for the activity of the DPM synthase [14,15]. DPM2 is associated with the DPM3 subunit via its N-terminus but does not interact directly with the catalytic subunit [14]. Subsequent studies showed that DPM2 regulates the activity of one more enzymatic complex. Watanabe et al. [18] have found that DPM2 enhances the activity of glucosylphosphatidylinositol-*N*-acetylglucosaminyl transferase (GPI-GnT), which transfers *N*-acetylglucosamine from UDP-*N*-acetylglucosamine to phosphatidylinositol, initiating the synthesis of GPI anchors for cell-surface proteins [19]. GPI-GnT is a complex glucosyltransferase of seven subunits: PIG-A, PIG-C, PIG-H, PIG-Q, PIG-P, PIG-Y, and DPM2 in humans [20]. Watanabe et al. [18]) have shown a coregulation of the DPM synthase and the GPI-GnT complex by DPM2. 

Notably, the DPM2 subunit has only been detected in the human class of DPM synthases, and no similar protein has been found in organisms with the *S. cerevisiae* type of the enzyme. 

In this study, we report that, in fact, the previously uncharacterized Yil102c-A protein of *S. cerevisiae* is homologous to DPMII from *Trichoderma*. Additionally, *dpm2* from *Trichoderma* suppresses the lethal phenotype of the *yil102c-A∆* mutant of *S. cerevisiae*, and Yil102c-A interacts physically with Dpm1 and Spt14, a homolog of PIG-A from humans. 

## 2. Results 

### 2.1. Identification and Cloning of YIL102c-A

Using the DPMII sequence from *Trichoderma reesei* as a probe, we found a homologous sequence in the *S. cerevisiae* genome denoted as the *YIL102C-A* gene.

**Figure 2:**
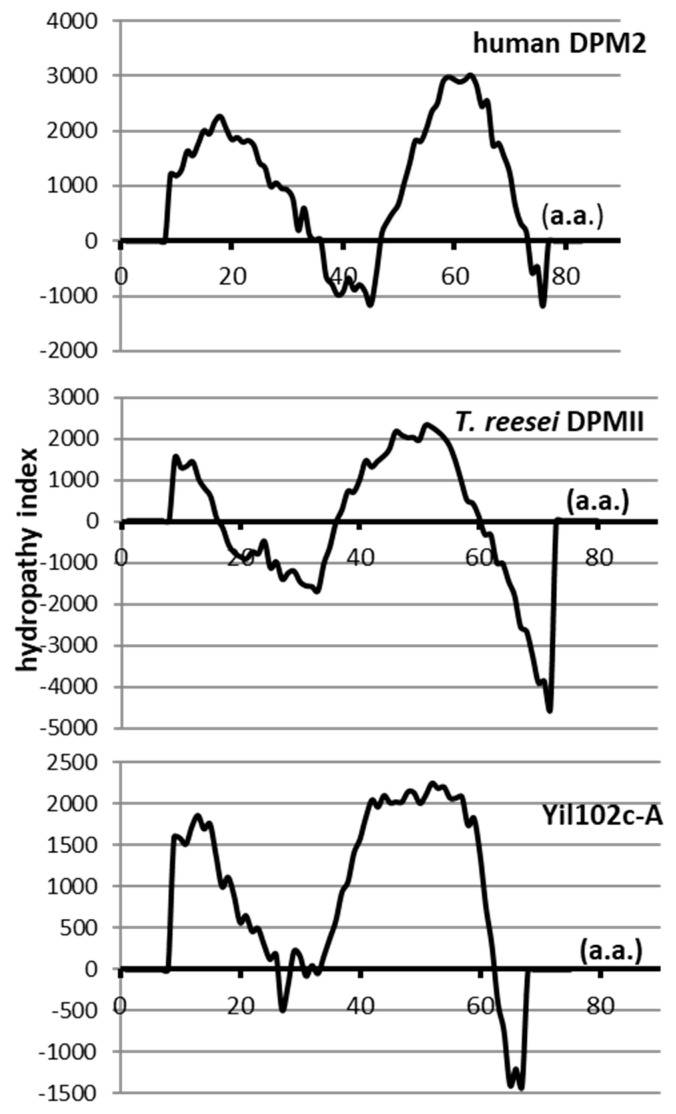
Hydropathy profiles of dolichyl phosphate mannose 2 (DPM2) from humans, *T. reesei*, and *S. cerevisiae*.

Its 228-bp open reading frame (ORF) encoded a putative protein of 75 amino acids, showing 36% identity and 66% similarity to the *Trichoderma reesei* DPMII protein (E value 1/10^14^), and 36% identity and 55% similarity to human DPM2 (E value 5/10^5^). No information about the gene or its product is available. Although the identity and similarity between Yil102c-A and *Trichoderma* DPMII or human DPM2 were low, all those proteins had similar hydropathy profiles and were predicted to comprise of two transmembrane regions (Figure 2). 

The Tmpred program [21] was used to determine the hydropathy profiles of the regulatory Dpm2 subunit of DPM synthases from humans and *T. reesei* and the putative protein Yil102c-A from *S. cerevisiae*. 

**Figure 3:**
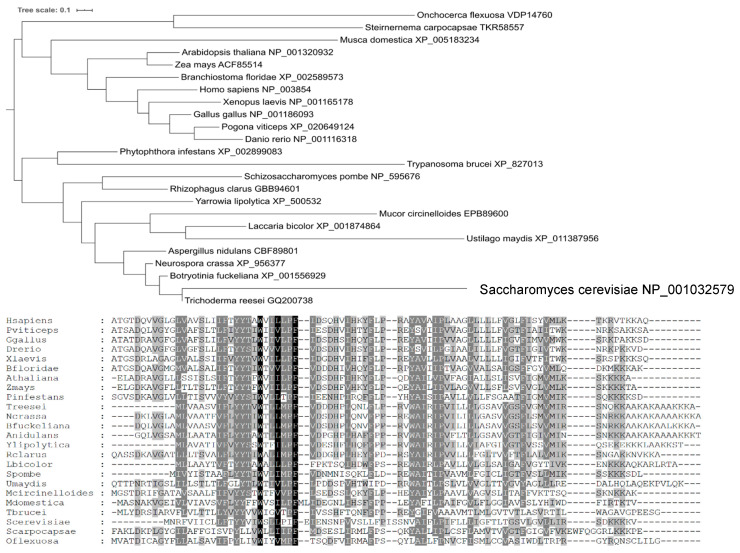
Phylogenetic tree and amino acid sequences alignment of DPM2 from selected organisms.

A phylogenetic analysis (Figure 3) showed that Yil102c-A is closely related to the DPMII proteins from filamentous fungi. 

Species names are followed by gene identifiers. Sequences were aligned with MAFFT v7.409 using the automate strategy [22] and trimmed manually. 

The predicted amino-acid sequence of Yil102c-A and its alignment with sequences of homologous proteins are shown below. 

Identical or similar amino acids present in all the organisms are marked in black; amino acids of the same chemical character found in most of the organisms are marked in dark gray; amino acids identical in only a few organisms are marked in light gray.

### 2.2. Lack of YIL102c-A is Lethal

**Figure 4:**
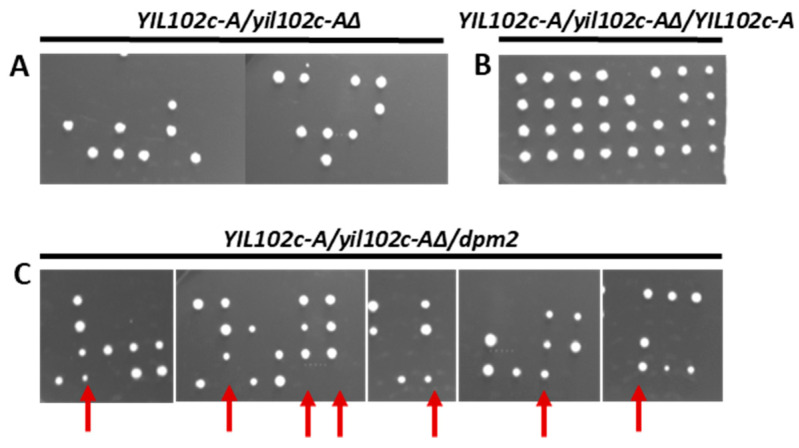
Effect of deletion of *YIL102c-A* gene and its reversal by ectopic expression of *YIL102c-A* or *Trichoderma dpm2*. Diploid *S. cerevisiae* strain BY4743 *YIL102c-A::kanMX4/YIL102c-A,* carrying an empty plasmid or expressing *YIL102c-A* or *Trichoderma dpm2,* was sporulated; tetrads were dissected, and spores allowed to germinate on medium without uracil. (**A**) (*YIL102c-A/yil102c-A∆*) BY4743 *YIL102c-A::kanMX4/YIL102c-A,* carrying an empty plasmid YEplac195; (**B**) (YIL102c-A/yil102c-A∆/YIL102c-A) BY4743 YIL102c-A::kanMX4/YIL102c-A, expressing YIL102c-A; (**C**) (YIL102c-A/yil102c-A∆/dpm2) BY4743 YIL102c-A::kanMX4/YIL102c-A, expressing dpm2 gene from *T. reesei*. Arrows indicate four and three viable spores that were further analyzed.

To examine the role of Yil102c-A in the yeast cells, we constructed an *S. cerevisiae* strain with a deletion of *YIL102c-A*. The diploid strain BY4743 was transformed, with the kanamycin cassette flanked by *YIL102c-A* sequences; the resultant *S. cerevisiae yil102c-A∆/YIL102c-A* strain was sporulated, and tetrads were dissected. Only two spores were viable, and both of them contained the intact *YIL102c-A* gene (Figure 4A). 

This result shows that the deletion of *YIL102c-A* is lethal. When the *YIL102cA::kanMX4/YIL102c-A* strain expressing *YIL102c-A* was sporulated, all four spores were viable (Figure 4B), indicating that *YIL102c-A* complemented the defect caused by the original *YIL102c-A* disruption. 

### 2.3. Complementation of YIL102c-A Deletion in S. cerevisiae by dpm2 Gene from Trichoderma 

The *S. cerevisiae yil102c-A∆/YIL102cA* mutant was transformed with the *dpm2* gene from *Trichoderma*, sporulated, and the tetrads were dissected and the spores cultivated on a selective medium without uracil. We found four viable spores, although the transformant expressing the *dpm2* gene from *Trichoderma* sporulated much worse (Figure 4C) than the *YIL102c-A* expressing strain (Figure 4B). Furthermore, colonies growing from spores containing the *dpm2* gene were smaller. The spore clones were then plated on a selective medium with kanamycin and without uracil, and the plasmid isolated from the growing strain was sequenced. The plasmid carried the *Trichoderma dpm2* gene, which confirmed that it could complement the *YIL102c-A* deletion. 

### 2.4. Comparison of Yil102c-A and DPMII Proteins

To verify that the DPMII protein from *Trichoderma* could fully substitute Yil102c-A in the *S. cerevisiae yil102c-A∆-dpm2* strain, we analyzed its growth kinetics and determined the activity of the enzymes predicted to cooperate with Yil102c-A based on its analogy with the human DPM2 protein. 

Strains were cultivated in SC medium with 2% glucose, without uracil or YPD medium, at 28 °C. 

**Figure 5:**
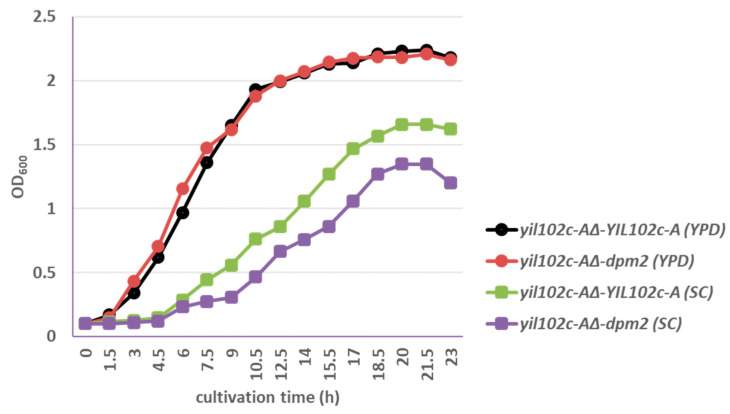
Growth of *S. cerevisiae* yil102c-A∆ carrying the YIL102c-A gene or dpm2 gene from *Trichoderma reesei*.

The *yil102c-A∆-dpm2* strain showed identical growth kinetics as *yil102c-A∆-YIL102c-A* when cultivated on YPD medium and slightly slower growth comparing to the *YIL102c-A*-expressing strain on SC medium (Figure 5). 

Strains were cultivated overnight, cells were homogenized, and the membrane fraction was obtained. Enzyme activity was analyzed using radiolabeled substrates, as described in the materials and methods section.

Data are mean ± standard deviation from five independent experiments, each determined in triplicate.

**Figure 6:**
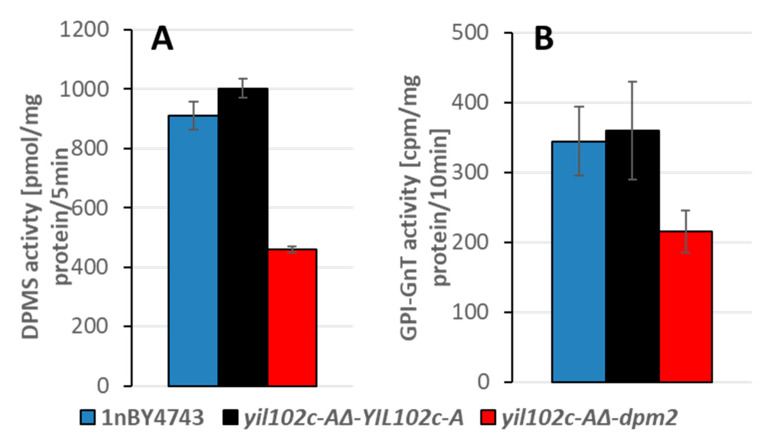
Activity of DPM synthase (**A**) and GPI-GnT (**B**) in the membrane fraction of *S. cerevisiae yil102c-A∆* carrying the *YIL102c-A* gene or *dpm2* gene from *Trichoderma reesei* and in the haploid 1nBY4743 strain derived from the BY4743 diploid strain after sporulation and tetrad dissection.

The activities of the DPM synthase and GPI-GnT, both regulated by DPM2 in humans, were significantly lower in the *yil102c-A∆-dpm2* strain than in the *YIL102c-A*-expressing strain (Figure 6 and Figure S3). 

### 2.5. Yil102c-A Interacts with DPM1 and Spt14 (PIG-A) 

Since the activity of the DPM synthase and GPI-GnT depended to some extent on the presence of the Yil102c-A protein, we checked if it interacts with Dpm1 and Spt14 (a PIG-A homolog), similar to what had been found for human DPM2. This was not obvious, bearing in mind that the DPM synthase in *S. cerevisiae* has been reported to be a single Dpm1 protein. 

For coimmunoprecipitation, Yil102c-A was tagged with the myc epitope on the N- or C-terminus to avoid the possibility that the tag could prevent the interaction with Dpm1 or Spt14. 

*S. cerevisiae* strains were cultivated in media with indicated supplements, and cell-free extracts were prepared. 

Yil102c-A tagged with the myc epitope at the N- or C-terminus was trapped on Myc-Trap Agarose beads, together with interacting proteins. Dpm1 and Spt14 were detected in the immunoprecipitates using specific antibodies, namely, cultivation with galactose-induced production of Yil102c-A tagged at the C-terminus and cultivation with Cu^2+^-induced N-terminally tagged Yil102c-A. 

**Figure 7:**
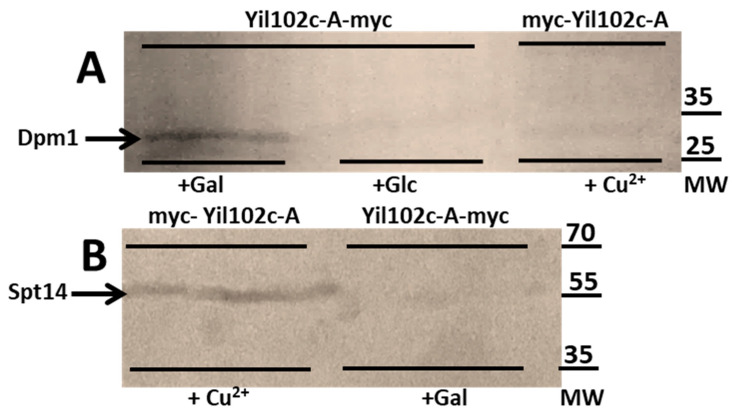
Immunodetection of Dpm1 (**A**) and Spt14 (**B**) bound to Yil102c-A.

As shown in Figure 7, both Dpm1 and Spt14 coimmunoprecipitated with Yil102c-A. Notably, Dpm1 was only found to interact with Yil102c-A tagged at the C-terminus and Spt14 with Yil102c-A tagged at the N-terminus, which suggested that the two proteins could interact with different fragments of Yil102c-A. 

## 3. Discussion

In this study, we have shown that the previously-uncharacterized Yil102c-A protein is a functional Dpm2 subunit of the DPM synthase in *S. cerevisiae*. 

The DPMII protein is a regulatory subunit of DPM synthase in *Trichoderma*, while in *S. cerevisiae*, the DPM synthase was reported to be a single fully functional Dpm1 protein [8,12,23] regulated by phosphorylation/dephosphorylation of serine 141 by cAMP-dependent protein kinase [24]. The same mode of regulation has been reported for human [25,26] and *Trichoderma* [13,27] enzymes despite them having the regulatory subunit DPMII/DPM2. This subunit is not essential in human cells, albeit the activity of DPM synthase and GPI-GnT was drastically reduced in a DPM2-null mutant [14]. 

In contrast, when the *Trichoderma dpm1* and *dpm3* genes were expressed in an *S. cerevisiae dpm1∆* strain, the full activity of the DPM synthase was restored despite the absence of the *Trichoderma* DPMII subunit [15]. That result indicated that the regulatory subunit of the *Trichoderma* DPM synthase was, in fact, dispensable or, much more likely, there is an unknown *S. cerevisiae* protein playing a role. 

Indeed, a search of the database of predicted *S. cerevisiae* proteins revealed a previously uncharacterized sequence, Yil102c-A, similar to that of DPMII from *T. reesei*. Deletion of the *YIL102c-A* gene turned out to be lethal, and this defect was reversed by the expression of the *dpm2* gene from *Trichoderma.* This is direct evidence that *S. cerevisiae* does have a homolog of DPMII protein and that Yil102c-A performs this function. The replacement of Yil102c-A by DPMII resulted in decreased activities of the DPM synthase and GPI-GnT, probably reflecting the fact that the activity of DPM synthase controlled by the DPMII subunit in *Trichoderma* is lower than the natural DPM synthase activity in *S. cerevisiae* [28]. 

As mentioned earlier, the expression of only two DPM synthase subunits (DPMI and DPMIII) from *Trichoderma* in yeast gave an activity identical to that typical for yeast, suggesting that the regulatory yeast subunit Yil102c-A boosted the activity of the *Trichoderma* synthase [15]. 

We also found that the replacement of Yil102c-A by DPMII reduced the GPI-GnT activity. It is not known how this activity develops in *Trichoderma*, but in human cells, it was reduced three-fold in the absence of DPM2 [18]. 

Our result showed that YIL102c-A influences the activities of the DPM synthase and GPI-GnT; therefore, we checked whether Yil102c-A interacts physically with the Dpm1 and Spt14 proteins. Such interactions have been described for the human DPM2 protein [14,18]. In accordance, coimmunoprecipitation confirmed that similar to yeast, Yil102c-A interacts with both Dpm1 and Spt14. The interaction between Yil102c-A and Dpm1 was abolished when the N-terminus of Yil102c-A was tagged with the myc epitope, suggesting that it is critical for the interaction. In the human DPM synthase, the DPM2 subunit, located in the ER membrane, interacts directly with DPM3, which, in turn, interacts with DPM1 [14]. Both DPM2 and DPM3 are hydrophobic. The situation in yeast is likely to be similar: the hydrophobic character of Yil102c-A enables its interaction with the C-terminal transmembrane domain of Dpm1, performing an anchoring role for the yeast DPM synthase that is analogous to that of DPM3 in the human enzyme. 

The association of DPM2 with human GPI-GnT is not fully understood. It was found that DPM2 associates with PIG-A, PIG-C, and PIG-Q in the membrane [18,20]. Sobering et al. [29] have proposed that in yeast, GPI-GnT is regulated by Ras2. GTP-bound Ras2 associates with the GPI-GnT complex in vivo and inhibits its activity. Our results indicate that Yil102c-A has an opposite, stimulatory effect. 

Our results showed that although the DPM synthase and the GPI transferase belong to different pathways (glycosylation and GPI anchor synthesis, respectively), they are physically linked by the same regulatory protein YIL102c-A, not only in humans but also in yeast. This makes additional sense if we consider that the synthase of the GPI anchor uses the product of the DPM synthase.

## 4. Materials and Methods

### 4.1. Strains and Growth Conditions

Two diploid strains of *S. cerevisiae* were used: BY4743 (Mat **a**/α; his3Δ1/his3Δ1; leu2Δ0/leu2Δ0; lys2Δ0/LYS2; MET15/met15Δ0; ura3Δ0/ura3Δ0) and W303-1b (Mat **a**/α; leu2-3,112 trp1-1 can1-100 ura3-1 ade2-1 his3-11,15). 

Yeast was grown in SC medium (synthetic complete medium: 0.67% yeast nitrogen base without amino acids; 2% glucose) [30] with necessary supplements or YPD (1% yeast extract, 2% bacto-peptone, 2% glucose) medium; for sporulation, 2% bacto-agar with 1% potassium acetate pH 7 was used. 

### 4.2. Deletion of YIL102c-A Gene in BY4743 Strain

A DNA fragment of about 1600 bp, containing a kanamycin resistance cassette flanked by *YIL102c-A* sequences, was amplified by PCR using a pFA6a-kanMX6 (Addgene, Watertown, MA, USA) [31] plasmid as a template, primers yilR and yilL (Table S1), and the following PCR conditions: 95 °C for 3 min, followed by 30 cycles of 95 °C for 30 s, 55 °C for 30 s, and 72 °C for 30 s, with a final extension step at 72 °C for 5 min. The PCR product was subjected to agarose gel electrophoresis, and the appropriate band was isolated and sequenced. This PCR product was then used for the transformation of the BY4743 strain. Transformants were selected on SC medium with kanamycin. 

The resultant *yil102c-A∆/YIL102c-A* strain was transformed with an expression plasmid carrying *YIL102c-A*. The 228-bp ORF with a 3′ UTR of 20 bp was obtained by PCR using the 5′UTR and LYILclo primers and the following PCR conditions: 95 °C for 3 min, followed by 30 cycles of 95 °C for 30 s, 55 °C for 30 s, and 72 °C for 30 s, with a final extension step at 72 °C for 5 min. The PCR product was ligated into the pGEM–T EasyVector kit (Promega, Medison, WI, USA), checked by sequencing after checking cut out with EcoRI, and ligated with the YEplac195 vector (SnapGene, San Diego, CA, USA) with the *URA3* marker. 

In conjunction, the *yil102c-A∆/YIL102c-A* strain was transformed with *dpm2* cDNA from *T. reesei*. *dpm2* was amplified on a *Trichoderma* cDNA bank using primers Udpm2Age and LdpmXho and was ligated into the AgeI/XhoI-cut YEplac195 vector. To obtain the PCR product, the same protocol was used as for the amplification of *YIL102c-A.*

Transformants of *S. cerevisiae yil102c-A∆/YIL102c-A* carrying YEplac195-YIL or YEplac195dpm2 were sporulated and the tetrads were dissected. Spores growing on medium with kanamycin and without uracil were used for further experiments. 

### 4.3. Overexpression of YIL102c-A-myc in S. cerevisiae 

For immunoprecipitation, *YIL102c-A* was tagged with the myc epitope and cloned into the YEp105myc_TRP1_CUP1 vector, simultaneously swapping out the ubiquitin-encoding ORF [32]. To this end, *YIL102c-A* was amplified on yeast genomic DNA using primers UYIL102 and LYILclo and the following PCR conditions: 95 °C for 3 min, followed by 30 cycles of 95 °C for 30 s, 55 °C for 30 s, and 72 °C for 30 s, with a final extension step at 72 °C for 5 min. The PCR product was cut with XhoI and ligated into the Ep105myc_TRP1_CUP1 vector cut with BglII, blunted with Klenow, and then cut with XhoI. The resultant YEp105mycYIL102c-A plasmid carries *YIL102c-A* tagged with the myc epitope on the N-terminus under the *CUP1* promoter (Figure S1). 

*YIL102c-A* tagged with the myc epitope on the C-terminus was amplified by PCR using primers Uyilsac and Lyilmyckpn, cut with SacI/KpnI, and cloned into the pYES2 vector under the *GAL1* promoter (Figure S1). These plasmids were used to overexpress *YIL102c-A*-myc in the W303 strain. 

Both PCR products were checked by sequencing. 

### 4.4. Membrane Preparation 

*S. cerevisiae* was cultured in YPD medium to OD_600_ = 1, harvested by centrifugation, and resuspended in 50 mM Tris-HCl, pH 7.4, containing 15 mM MgCl_2_ and 9 mM β-mercaptoethanol. Cells were homogenized in a bead beater with 0.5-mm glass beads, and the homogenate was centrifuged at 5000× *g* for 10 min to remove cell debris and unbroken cells. The supernatant was then centrifuged at 100,000× *g* for 1 h. The membrane pellet was homogenized in 50 mM Tris-HCl, pH 7.4, containing 3.5 mM MgCl_2_ and 6 mM β-mercaptoethanol, and used as the source of enzymes. The whole procedure was performed as described before [33].

### 4.5. Protein Determination

Protein concentration was determined using Folin phenol reagent, according to Lowry et al. [34]. 

### 4.6. Dolichyl Phosphate Mannose Synthase (EC 2.4.1.83) Activity

DPM synthase activity was analyzed in the membrane fraction (100 µg of membrane protein) by incubating it with 8 × 10^4^ cpm of GDP [^14^C]mannose (sp. act. 55 Ci/mol, American Radiolabeled Chemicals Inc., St. Louis, MO, USA) and 5 ng of dolichyl phosphate (Dol-P; a kind gift from Prof. Ewa Swiezewska of the Institute of Biochemistry and Biophysics, Polish Academy of Sciences, Warsaw, Poland) in 40 mM Tris-HCl buffer, pH 7.4, with 10 mM MgCl_2_ and 0.1% Nonidet P-40 for 5 min at 30 °C in a total volume of 50 µL [13,35]. The reaction was stopped by the addition of 4 mL of chloroform:methanol (3:2, *v*/*v*). The excess of GDP mannose was washed out from the organic fraction by the addition of 1 mL of 4 mM MgCl_2_. The upper phase was removed, and the organic lower phase was dried. Radioactive dolichyl phosphate mannose was measured in the organic phase in a scintillation counter. 

### 4.7. Glucosylphosphatidylinositol-N-Acetylglucosaminyl Transferase (GPI-GnT) (EC 2.4.1.198) Activity

GPI-GnT activity was analyzed in the membrane fraction (100 µg of membrane protein) by incubating it with 175 × 10^4^ cpm of UDP[^14^C]GlcNAc (sp. act. 55 Ci/mol, American Radiolabeled Chemicals Inc., St. Louis, MO, USA) in 100 mM Tris-HCl, pH 7.5, with 1 mM EGTA, 3 mM (CH_3_COO)_2_Mg, 0.5 mM MnCl_2_, 1 mM CoA, 1 mM ATP, 0.2 µg/mL tunicamycin, and 100 µg of membrane protein. After 30 min of incubation at 30 °C in a total volume of 100 μL, the reaction was stopped by the addition of 1.5 mL of chloroform:methanol (1:2, *v*/*v*), mixed, and centrifuged. The supernatant was collected and the pellet extracted again with 0.5 mL of chloroform:methanol. The organic phases were pooled, dried, and partitioned between 1 mL of water and 1 mL of butanol to remove the salt. The butanol phase was dried and dissolved in 20 µl of chloroform:methanol (1:2, *v*/*v*). Lipids were separated by TLC on silica gel 60 in chloroform:methanol:25% NH_4_OH:1 M CH_3_COONH_4_:water (180:140:11:8:21, *v*/*v*/*v*/*v*). After development, the plates were air-dried, and the radiolabeled early precursors of GPI were visualized in a FLA7000 phosphoimager, scraped, and measured using a scintillation counter.

### 4.8. Coimmunoprecipitation was Done Using Myc-Trap Agarose kit 

To induce the expression of myc-tagged Yil102c-A, *S. cerevisiae* carrying *myc-YIL102c-A* under the *CUP1* promoter (Figure S1) was cultivated overnight in 50 mL SC medium supplemented with 0.2 mM CuSO_4_ and *S. cerevisiae* carrying *YIL102c-A-myc* under the *GAL1* promoter (Figure S1) was cultivated overnight in SC medium with galactose. Yeast was then homogenized in a bead beater, with 0.5-mm glass beads in lysis buffer with 1 mM PMSF, and the homogenate was centrifuged at 5000× *g* for 10 min to remove cell debris and unbroken cells. Cell-free extract (500 μg protein) containing tagged Yil102c-A with associated proteins (Dpm1 or Spt14) was incubated at 4 °C with 25 μL of Myc-Trap Agarose (ChromoTek, Planegg, Germany) beads washed twice with 500 μL of ice-cold dilution buffer, according to the producer’s manual for immunoprecipitation of proteins from mammalian cell extract. After 24 h, the beads were centrifuged at 2500× *g* for 2 min. After washing twice in the ice-cold dilution buffer (enclosed with the kit), the beads were melted (10 min at 95 °C) and the released proteins subjected to SDS PAGE, transferred on an Immobilon P membrane (Millipore, Billerica, MA, USA) and incubated overnight with antibodies against Dpm1 (Thermo Fisher Scientific) or PIG-A (homolog of Spt14; Santa Cruz Biotechnology, Dallas, TX, USA). The bound antibodies were detected using appropriate secondary antibodies conjugated with alkaline phosphatase (anti-mouse IgG for detection of Dpm1, anti-rabbit IgG for Spt14; both from Sigma-Aldrich, St. Louis, MO, USA). 

The presence of Dpm1, Spt14, and myc-tagged Yil102c-A proteins in the cell-free extracts and on Myc-Trap beads was first checked on dot blots using specific antibodies (Figure S2).

### 4.9. Phylogenetic Analysis

Selection of Dpm2 dataset: sequences of well-characterized human DPM2 (Acc. no. NP_003854) and *Trichoderma reesei* DPMII (Acc. no. ACS74782) were queried against the GenBank nonredundant protein database using BLAST [36]. All proteins with the PF:07297 Pfam domain of Dpm2 were extracted and subjected to further analysis. Each taxonomic group was represented by at least one species. Sequences were aligned with MAFFT v7.409 using the automate strategy [22] and trimmed manually. The phylogenetic tree was computed with PhyML v3.0 using the automatic model selection criterion SMS [37] and the Bayesian information criterion. The phylogenetic tree was generated using the iTol website [38].

## 5. Conclusions

Deletion of the *YIL102c-A* gene in the yeast *S. cerevisiae* is lethal, and the *dpm2* gene from *T. reesei* can complement this deletion. The replacement of Yil102c-A with DPMII affects the activity of the DPM synthase and GPI-GnT. In addition, Yil102c-A interacts physically with Dpm1 and Spt14, to how DPM2 interacts with the DPM1 and PIG-A proteins in human cells. This is a new finding in lower eukaryotic species, especially in yeast. 

All these data indicate that Yil102c-A is a functional regulatory subunit of the DPM synthase in *S. cerevisiae*.

## Supplementary Materials

Supplementary materials can be found at https://www.mdpi.com/1422-0067/21/23/8938/s1.
