# Supplementary material for: Yil102c-A is a Functional Homologue of the DPMII Subunit of Dolichyl Phosphate Mannose Synthase in Saccharomyces cerevisiae
Source: Int J Mol Sci. 2020 Nov 25;21(23):8938. doi: 10.3390/ijms21238938 (PMC7728079; doi:10.3390/ijms21238938)
Supplement: Supplementary file 1 [file ijms-21-08938-s001.pdf]

Table S1. Primers used in this study.

Primer Name	5′-sequence -3′	Purpose	Remarks
yilF	ATGAACCGTTTTGTAATTATTTGCCT TCTTTTTACTTATTATGTCATATGcgta cgctgcaggtcgac	Disruption of *YIL102cA*	Capital letters –fragment homologous to *YIL102cA* Small letters - fragment homologous to kanamycin cassette
yilR	TTAAACTTTTTTCTTTTTATCGCTGC GTATAAGCAATACACCTAAAACAG AACtcgatgaattcgagctcg		
5'UTR	CTGCAGCTGTTAAACATGGGCCTGT AAGCG	Amplification of *YIL102cA*	PCR product was cloned to pGEM-T Easy vector, cut out with EcoRI and ligated to YEp195lac-URA
LYILclo	GTGACTCGAGCCTCTTAAGGCTTAG		
Udpm2Age	ACCGGTTGGGCATAGTAAACGTCTG TTTAGTTTCATGCTAGTTGCTGCTTC CGTCATCT	Amplification of *dpm2*cDNA from *T. reesei*	AgeI restriction site underlined PCR product was ligated to YEplac195-URA
LdpmXho	CTCGAGCCTCTTAAGGCTTAGATTA CGCCTTCTTCTTGGCGGCGGCCT		XhoI restriction site underlined
UYIL102	ATGAACCGTTTTGTAATTATTTGCCT TCTTTTTAC	Used with LYILclo for amplification of *YIL102c-A*	Cloned to YEp105myc_TRP1 _CUP1
Uyilsac	TGAGCTCATGAACCGTTTTGTAATT ATTTG	Amplification of YIL102c-A with myc tag at the C-terminus (small letters)	PCR product cloned to pYES2 vector SacI and KpnI restriction sites underlined
Lyilmyckpn	TGGTACCcaagtcttcttcagaaataagcttttgttc AACTTTTTTCTTTTTATCGCTGCGT		

Supplementary figure

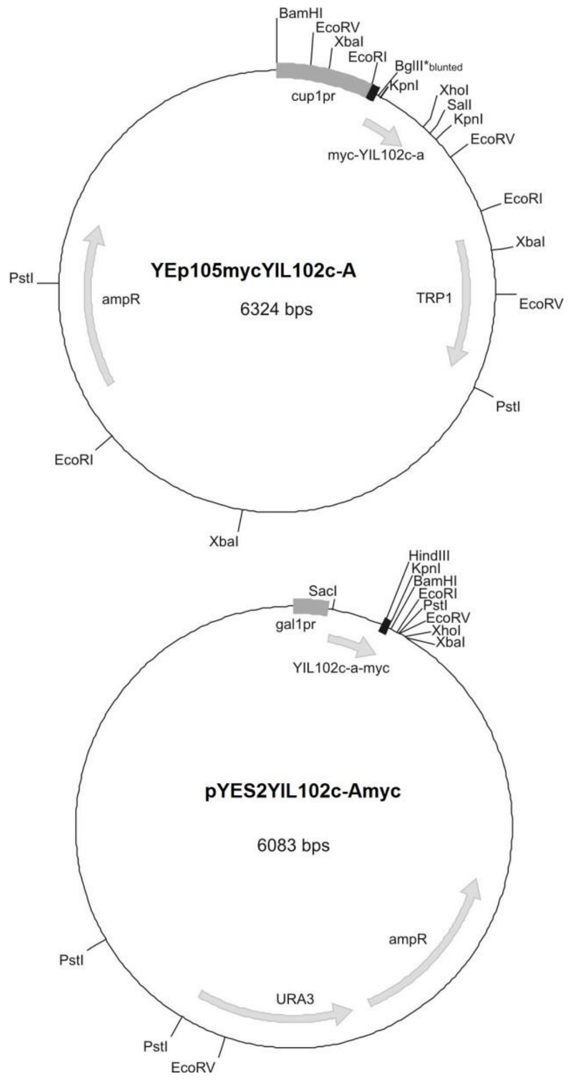

Figure S1. Expression plasmids encoding Yil102-cA protein with myc epitope on the N-terminus (YEp105mycYIL102c-A) and on the C-terminus (pYES2YIL102c-Amyc).

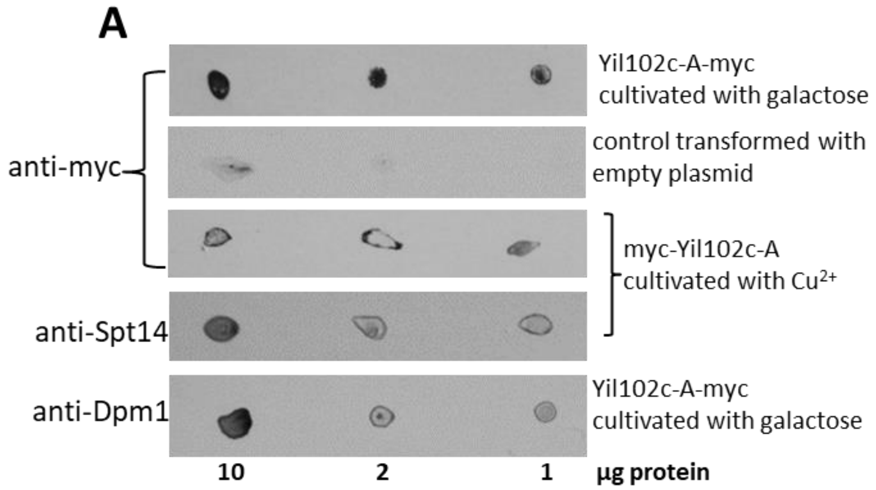

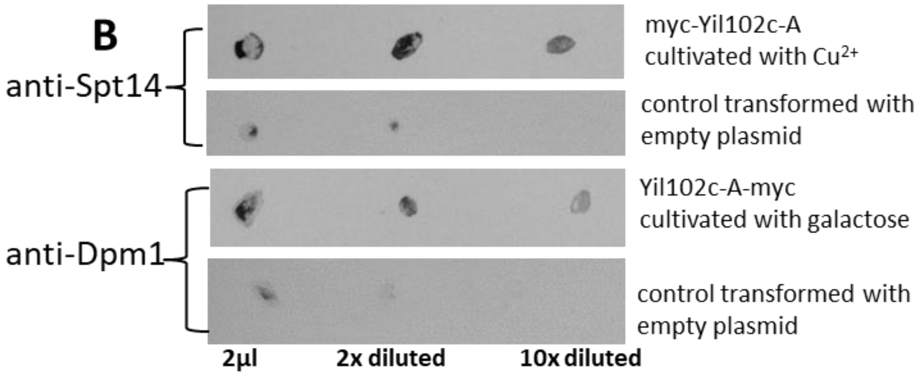

Protein concentration in the cell free extracts	
Yil102c-A-myc cultivated with galactose	13 µg/µl
myc-Yil102c-A cultivated with Cu^{2+}	14 µg/µl
Control transformed with empty plasmid pESC URA	25 µg/µl

Figure S2. Immunodetection of myc-tagged Yil102c-A, Spt14 and Dpm1 in the cell free extracts (A) and in Myc-Trap beads (B) using anti-myc, anti-PigA (Spt14) and anti-Dpm1 antibodies.

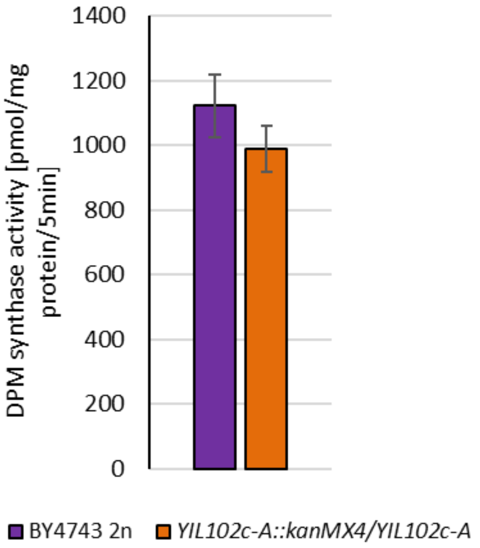

Figure S3. Activity of DPM synthase in membrane fraction od *S. cerevisiae* diploid strain *YIL102c-A::kanMX4/YIL102c-A* carrying one deleted copy of *YIL102c-A* gene compared to the parental diploid strain BY4743 2n. .
